# Device-measured physical activity and sedentary time in a national sample of Luxembourg residents: the ORISCAV-LUX 2 study

**DOI:** 10.1186/s12966-022-01380-3

**Published:** 2022-12-29

**Authors:** Paul J. Collings, Anne Backes, Gloria A. Aguayo, Laurent Malisoux, on behalf of the ORISCAV-LUX study group, Ala’a Alkerwi, Stephanie Noppe, Charles Delagardelle, Jean Beissel, Anna Chioti, Saverio Stranges, Jean-Claude Schmit, Marie-Lise Lair, Marylène D’Incau, Jessica Pastore, Gwenaëlle Le Coroller, Brice Appenzeller, Sophie Couffignal, Manon Gantenbein, Yvan Devaux, Michel Vaillant, Laetitia Huiart, Dritan Bejko, Torsten Bohn, Hanen Samouda, Guy Fagherazzi, Magali Perquin, Maria Ruiz, Isabelle Ernens

**Affiliations:** 1 Physical Activity, Sport and Health Research Group, Department of Precision Health, Luxembourg Institute of Health, 1A-B, rue Thomas Edison, Strassen, L-1445 Luxembourg; 2 Deep Digital Phenotyping Research Unit, Department of Precision Health, Luxembourg Institute of Health, 1A-B, rue Thomas Edison, Strassen, L-1445 Luxembourg

**Keywords:** Inactivity, Sitting, Correlates, Adults, Demographic factors, Socioeconomic factors, Behaviour, Lifestyle, Health status, Obesity

## Abstract

**Background:**

Existing information about population physical activity (PA) levels and sedentary time in Luxembourg are based on self-reported data.

**Methods:**

This observational study included Luxembourg residents aged 18-79y who each provided ≥4 valid days of triaxial accelerometry in 2016-18 (*n*=1122). Compliance with the current international PA guideline (≥150 min moderate-to-vigorous PA (MVPA) per week, irrespective of bout length) was quantified and variability in average 24h acceleration (indicative of PA volume), awake-time PA levels, sedentary time and accumulation pattern were analysed by linear regression. Data were weighted to be nationally representative.

**Results:**

Participants spent 51% of daily time sedentary (mean (95% confidence interval (CI)): 12.1 (12.0 to 12.2) h/day), 11% in light PA (2.7 (2.6 to 2.8) h/day), 6% in MVPA (1.5 (1.4 to 1.5) h/day), and remaining time asleep (7.7 (7.6 to 7.7) h/day). Adherence to the PA guideline was high (98.1%). Average 24h acceleration and light PA were higher in women than men, but men achieved higher average accelerations across the most active periods of the day. Women performed less sedentary time and shorter sedentary bouts. Older participants (aged ≥55y) registered a lower average 24h acceleration and engaged in less MVPA, more sedentary time and longer sedentary bouts. Average 24h acceleration was higher in participants of lower educational attainment, who also performed less sedentary time, shorter bouts, and fewer bouts of prolonged sedentariness. Average 24h acceleration and levels of PA were higher in participants with standing and manual occupations than a sedentary work type, but manual workers registered lower average accelerations across the most active periods of the day. Standing and manual workers accumulated less sedentary time and fewer bouts of prolonged sedentariness than sedentary workers. Active commuting to work was associated with higher average 24h acceleration and MVPA, both of which were lower in participants of poorer self-rated health and higher weight status. Obesity was associated with less light PA, more sedentary time and longer sedentary bouts.

**Conclusions:**

Adherence to recommended PA is high in Luxembourg, but half of daily time is spent sedentary. Specific population subgroups will benefit from targeted efforts to replace sedentary time with PA.

**Supplementary Information:**

The online version contains supplementary material available at 10.1186/s12966-022-01380-3.

## Introduction

Physical activity (PA) is favourably associated with physical and psychosocial health [1], and high sedentariness is detrimental [2]. New guidelines provided by the World Health Organization (WHO) recommend that adults perform 150-300 min of moderate intensity PA, or 75–150 min of vigorous intensity PA, or an equivalent combination of moderate-to-vigorous intensity PA (MVPA) per week. It is no longer required that MVPA is accumulated in at least 10 min bouts. There are no specific recommendations for sedentary time, other than it should be limited, and that replacing sedentary time with PA of any intensity is preferable [3].

Several studies have investigated the levels and correlates of leisure time or total MVPA, sedentary time, and specific sedentary behaviours (primarily TV viewing) in population-based samples of adults. Potential correlates include demographic, sociocultural, environmental, physical, and psychological factors [4, 5]. Comparatively few descriptive studies have investigated patterns of sedentary time accumulation, despite evidence that prolonged sedentary bouts may be particularly deleterious to cardiometabolic health [6]. In addition, relatively few studies have focussed on light PA, even though it is the biggest contributor to PA energy expenditure [7]. Replacing sedentary time with light PA also reduces cardiometabolic disease and mortality risk [8], and it may be easier to encourage participation in lighter than higher intensity PA since it is less strenuous and more accessible [9]. Additional studies are required to investigate the entire intensity spectrum of device-measured movement behaviours, including sedentary time patterns. This knowledge can be used to help guide policy formulation, and to assist public health experts in designing effective interventions that are tailored to the needs of specific groups.

This study was conducted to describe for the first time the volume and pattern of device-measured movement behaviours performed by adults living in the Grand Duchy of Luxembourg. The smallest country that is not a European microstate or island, Luxembourg is the wealthiest country in the world by gross domestic product per capita [10]. We aimed to investigate the sociodemographic, employment, and health-related correlates of movement behaviours, spanning the full intensity spectrum, and including markers of sedentary time accumulation. We further sought to supplement the device-measured data with contextual information about sport and exercise participation, to help shed light on the reasons for movement behaviour patterns. Finally, we estimated the national prevalence of meeting the revised WHO guideline for adult PA, which Luxembourg officially adopted in 2021 [11].

## Methods

### Study population

Data were from the ORISCAV-LUX 2 study, a national cross-sectional survey of cardiovascular risk factors in the Luxembourgish adult population [12]. In total, 1558 participants were enrolled to the study in 2016-18 and detailed information about demographic, economic, lifestyle, medical and health factors were collected. Approximately one-fifth of participants opted out of wearing an accelerometer (*n*=345), and after excluding participants with insufficient accelerometer data (*n*=76), or missing covariate information (*n*=15; mainly education level was missing), 1122 participants remained for this complete-case analysis (72% of the starting sample). Study approval was granted by the National Research Ethics Committee (N° 201.505/12) and all participants provided written informed consent.

### Physical activity and sedentary time metrics

Movement data were collected with an Actigraph GT3X+ accelerometer (Florida, USA), which was continuously worn on the wrist of the non-dominant hand (except when showering and during water activities) for one week. Data were sampled at a frequency of 30 Hz, and after download via the Actilife software (v6.13.3, Florida, USA) and calibration with the open-source R package *GGIR* (version 2.2-0), raw acceleration signals were averaged over 5s epochs [13]. All days with ≥10h of waking data were considered useable and a valid accelerometer wear period comprised ≥4 valid days of data (including ≥1 weekend day). From the 24h time-series, the average daily acceleration (m*g*; indicative of PA volume), intensity gradient (intensity distribution), and numerous Mx metrics were calculated [14]. The latter represent the average acceleration value above which the most active ‘x’ minutes of the day were accumulated. Sleep onset and waking times were detected using a validated method and the total sleep period was calculated [15]. Thereafter, validated thresholds were applied to estimate the average daily awake time that participants spent sedentary, in light PA and MVPA [16, 17]. Patterns of sedentary time accumulation were examined by calculating the median bout length, the number of prolonged sedentary bouts (≥30 min in duration), and the power-law exponent alpha. Power-law exponent alpha describes the distribution of bouts relative to their duration (lower values indicate a greater proportion of longer bouts). We further calculated the proportion of total sedentary time that was accumulated in ≥60 and ≥120 min bouts. Full details of the objective monitoring procedure and summary variables are available elsewhere [18, 19].

### Adherence to physical activity guidelines and participation in sport and exercise

To estimate the weekly MVPA volume, each participant’s activity record was extrapolated to represent five weekdays and two weekend days. The nature of the extrapolation depended on the extent and pattern of missing days. However, for the vast majority (98.1% of participants contributed four valid weekdays and an entire weekend of data) it entailed multiplying the total MVPA volume accumulated over weekdays by 1.25 (to account for one missing weekday), and adding this value to MVPA accumulated at the weekend. Following the extrapolation procedure, participants were classified as meeting the recommended volume of PA if they accumulated ≥150 min MVPA/week: 1) irrespective of bout length (the new international guideline), and 2) in bouts >10 min (the former international guideline) for comparison [3]. To provide contextual information, participants reported if they usually performed sport or exercise (yes / no), and the kinds of activities that they performed (free-text).

### Independent variables: Sociodemographic, occupational and health-related factors

Residential addresses were used to classify participants as living in one of three geographical districts (Luxembourg / Diekirch / Grevenmacher). Participants self-reported their sex (male / female) and age. The latter was categorised (25-34y / 35-44y / 45-54y / 55-64y / ≥65y). Participants provided information about their highest educational qualification, which was harmonised into three categories (higher education / high school / no diploma), and their current and former smoking habits (never / former / current smoker). Whether or not participants were in paid employment and their occupational PA level was captured. The responses were combined to indicate work type (sedentary / standing / manual (including heavy manual) / not in paid employment). Not in paid employment chiefly comprised retirees and homemakers but also included students and the unemployed. Commuters provided information about their primary mode of travel to work which was synthesised into three groups (motor vehicle / public transport / active travel). Self-rated general health was graded using a five-point Likert scale (excellent / very good / good / fair / poor), but because there were few observations in the extreme categories, the data were pooled with neighbouring groups (very good / good / fair). Weight and height were measured by trained personnel using standard procedures and calibrated equipment. The data were used to calculate body mass index (BMI, kg/m^2^) and to classify participants as normal weight (<25 kg/m^2^), overweight (≥25 to <30 kg/m^2^), or obese (≥30 kg/m^2^). Time-stamped information from accelerometers were used to denote the meteorological season of measurement (summer (June to August) / autumn (September to November) / winter (December to February) / spring (March to May)).

### Statistical analysis

Chi-square tests were used to compare study characteristics between men and women, and to compare adherence to the PA guidelines across age and sex strata. For the primary analysis, linear regression models were used to quantify the volume and pattern of PA and sedentary time (separate models were specified for each dependent variable). With the exception of mode of transport to work, all independent variables were simultaneously included in models to adjust for each other. To assess the association between mode of transport to work with dependent variables, models were subsequently rerun in a sub-sample of participants who were in paid employment and who commuted to work (*n*=707), with mode of transport included as an additional independent variable. The main results are presented as estimated marginal means with 95% confidence intervals (CI). To improve the normality of residual plots, the data for MVPA and all Mx metrics were natural log-transformed prior to analyses. The results have been back-transformed to original units. To explore factors that were related to the likelihood of habitually performing sport or exercise, logistic regression models were used to calculate odds ratios (OR). Logistic models were specified as per linear models. The supplementary material includes: (1) the results for district, which are not presented here because following a reorganisation of administrative divisions, Luxembourg is now divided into 12 administrative cantons; (2) The results for season because we did not possess repeated data collected on the same participants over a calendar year; (3) The results stratified by sex. All of the data were weighted (by district, sex and age based on STATEC census data [20]) to achieve nationally representative estimates. Analyses were performed using Stata 15.1. Statistical significance was set at *p*<0.05, but we place emphasis on the range of plausible values of associations, as indicated by CIs [21].

## Results

Table 1 provides a description of the 1122 study participants who together contributed 6718 valid days of accelerometry. The median (iqr) monitoring time was 1440 (1435 to 1440) min/d, and the mean age of participants was 48.4 (95% CI: 47.9 to 48.9) years. The vast majority of men who were not in paid employment were retired (82.2%), whereas half of unemployed women were retired (54.0%) and one-third were unpaid homemakers (34.0%). The mean BMI of the whole sample was 25.8 (95% CI: 25.6 to 26.1) kg/m^2^.

**Table 1:** Descriptive statistics of the study sample

	Men (*n*=523, 50.0)	Women (*n*=599, 50.0)	All (*n*=1122)	***p***-sex difference
District				
Luxembourg	360 (73.4)	432 (73.6)	792 (73.5)	
Diekirch	88 (14.8)	87 (14.5)	175 (14.7)	
Grevenmacher	75 (11.8)	80 (11.9)	155 (11.8)	0.95
Age group				
25-34y	65 (21.4)	65 (21.6)	130 (21.5)	
35-44y	117 (24.3)	133 (23.7)	250 (24.0)	
45-54y	136 (23.5)	167 (22.2)	303 (22.8)	
55-64y	128 (16.8)	151 (16.2)	279 (16.5)	
≥65y	77 (14.0)	83 (16.3)	160 (15.2)	0.39
Education level				
Higher education	239 (48.3)	243 (44.9)	482 (46.6)	
High school	215 (40.2)	264 (40.7)	479 (40.4)	
No diploma	69 (11.5)	92 (14.4)	161 (13.0)	0.27
Work type				
Sedentary	245 (48.6)	249 (43.1)	494 (45.9)	
Standing	55 (11.6)	95 (16.7)	150 (14.1)	
Manual	56 (12.0)	28 (5.0)	84 (8.5)	
Not in paid employment	167 (27.8)	227 (35.2)	394 (31.5)	<0.001
Mode of transport to work				
Motor vehicle	270 (54.8)	279 (49.1)	549 (52.0)	
Public transport	43 (9.0)	47 (8.2)	90 (8.6)	
Active travel	33 (6.7)	35 (5.6)	68 (6.1)	
No commute	177 (29.5)	238 (37.1)	415 (33.3)	0.07
Smoking status				
Never	280 (55.1)	385 (65.0)	665 (60.1)	
Former	170 (30.8)	149 (23.5)	319 (27.1)	
Current	73 (14.1)	65 (11.5)	138 (12.8)	0.006
Self-rated health				
Very good	195 (38.7)	195 (33.8)	390 (36.32)	
Good	274 (51.2)	335 (54.9)	609 (53.1)	
Fair	54 (10.1)	69 (11.3)	123 (10.7)	0.28
Weight category				
Normal	192 (39.6)	325 (57.7)	517 (48.6)	
Overweight	215 (40.8)	179 (28.2)	394 (34.5)	
Obese	116 (19.6)	95 (14.1)	211 (16.9)	<0.001
Season				
Summer	101 (20.5)	156 (25.7)	257 (23.1)	
Autumn	129 (24.2)	174 (29.4)	303 (26.8)	
Winter	161 (29.8)	136 (22.9)	297 (26.3)	
Spring	132 (25.5)	133 (22.0)	265 (23.8)	0.011
Performs sport or exercise	291 (56.0)	360 (60.8)	651 (58.4)	0.12

The data are observed *n* (weighted %) and sex comparisons were performed using Chi^2^ tests. Public modes of transport to work included journeys by bus (65.1%), train (27.8%), or both (7.1%). Active travel modes comprised walking (80.1%) and bicycling (19.9%). The mean travel time to work (and back) by mode of transport was 53.2 (95% CI: 50.4 to 56.0) min/d by motor vehicle, 81.2 (71.6 to 90.8) min/d by public transport, and 28.0 (21.6 to 34.3) min/d by active travel. Very good self-rated health included 58 participants who were in excellent health. Fair self-rated health included 14 participants who were in poor health

**Fig. 1:**
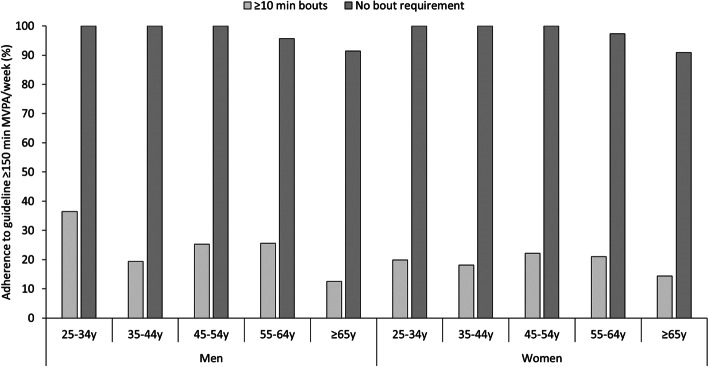
Adherence to the former (≥150 min MVPA/week in bouts ≥10 min) and current (≥150 min MVPA/week irrespective of bout duration) physical activity guidelines for adults, stratified by sex and age group. Data are weighted proportions

An unadjusted summary of movement behaviours revealed that nearly one-third of participants’ daily time was spent asleep (mean (95% CI): 7.7 (7.6 to 7.7) h/day), 51% was sedentary time (12.1 (12.0 to 12.2) h/day,), 11% of each day was light PA (2.7 (2.6 to 2.8) h/day) and 6% was MVPA (1.5 (1.4 to 1.5) h/day). The average 24h acceleration was 26.6 (26.2 to 27.1) m*g*. Participants performed on average 5.6 (5.5 to 5.7) bouts of prolonged sedentariness per day, and the median sedentary bout length was 32.0 (31.7 to 32.3) min. The median weekly volume of all MVPA was 583.0 (560.9 to 605.0) min/week, compared to just 48.4 (41.7 to 55.1) min/week when including only bouts >10 min. Figure 1 reveals there was complete adherence to the new PA guideline for the three youngest age groups, and adherence was lower (but still >90%) for the older ages (*p*<0.001). Overall, 98.1% of the study population met the new PA guideline and there was no difference in compliance between men and women (*p*=0.97). Adherence to the former PA guideline was much lower (21.9%) and higher in men than women (24.5% versus 19.2%, *p*=0.048).

**Fig. 2:**
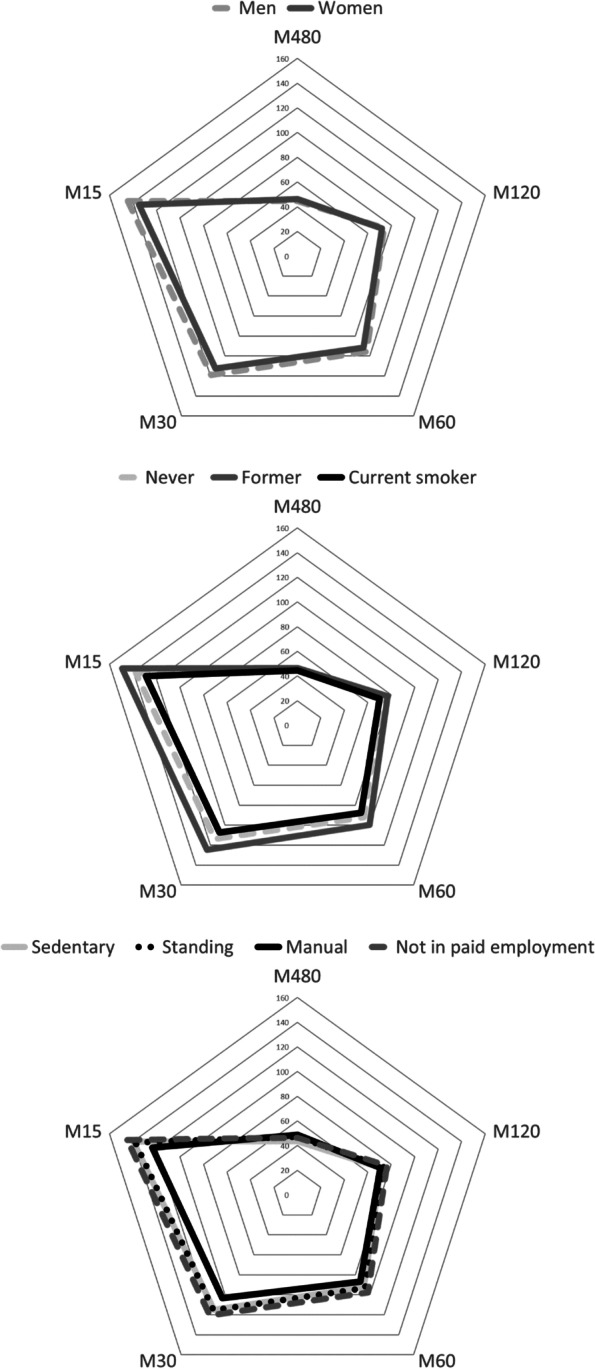
Radar plots illustrating Mx metrics that represent the acceleration above which the most active 480 min (one-third), 120 min, 60 min, 30 min, and 15 min of the day were accumulated, stratified by sex (top), smoking status (middle), and work type (bottom). The data are weighted estimated marginal means and represent the average acceleration above which the most active ‘x’ min of the day were performed (minutes did not need to be performed continuously or in bouts). For example, women exceeded 134.8 mg for a total of 15 min across the day, and men exceeded 144.4 mg for a total of 15 min across the day. Full results including 95% confidence intervals and *p*-values are presented in Table S3

Table 2 summarises the marginal mean estimates for the average 24h acceleration, intensity distribution and awake-time PA levels. The average 24h acceleration and time spent in light PA were higher in women than men. Relative to the youngest age group, participants aged 55-64y and ≥65y registered lower average 24h acceleration and less time in MVPA. Participants of lower education registered a higher average 24h acceleration and accumulated more intensity-specific PA, as did participants with standing and manual occupations compared to a sedentary work type. Both the average 24h acceleration and time spent in MVPA were lower in participants of poorer self-rated health and participants of higher weight status. Obese participants further performed less light PA. Relative to commuting by motor vehicle, active travel modes were associated with a higher average 24h acceleration and with more time spent in MVPA. Although time in MVPA did not differ by sex or smoking status, 24h intensity gradients were shallower in men than women and shallower in former smokers than never smokers. Figure 2 illustrates that these differences corresponded to higher average accelerations across the most active 30 min and 15 min of the day in men compared to women, and higher average accelerations across the most active periods of the day in former smokers compared to never smokers. Manual workers were characterised by a steeper intensity gradient, which corresponded to them achieving lower average accelerations across the most active 30 min and 15 min of the day than sedentary workers. Tables S1-S5 provide full details of the results for Mx metrics, and all results stratified by men and women. There was substantial overlap in most confidence intervals indicating that results did not substantively differ between sexes. However, men aged ≥65y performed more light PA than men in the youngest age group. There was also some indication that men who traveled to work by public transport spent more time in MVPA, and that not being in paid employment was associated with higher 24h acceleration in women. Men without a diploma were characterised by a steeper 24h intensity gradient. This corresponded to them achieving lower average accelerations across the most active 30 min and 15 min of the day.

**Table 2:** Volume and pattern of whole day (24h) acceleration and intensity distribution and awake-time intensity specific physical activity levels

	Average 24h acceleration (m***g***)	***p***-value	24h Intensity gradient	***p***-value	Light PA (min/d)	***p***-value	MVPA (min/d)	***p***-value
Sex								
Men	26.2 (25.5 to 26.8)	Ref	-2.58 (-2.60 to -2.56)	Ref	150.1 (146.4 to 153.7)	Ref	76.9 (73.6 to 80.2)	Ref
Women	**27.1 (26.5 to 27.7)**	**0.042**	**-2.65 (-2.66 to -2.63)**	**<0.001**	**174.4 (170.7 to 178.1)**	**<0.001**	79.8 (76.6 to 83.2)	0.22
Age group								
25-34y	28.1 (27.0 to 29.3)	Ref	-2.52 (-2.55 to -2.49)	Ref	158.9 (152.7 to 165.2)	Ref	93.3 (87.2 to 99.8)	Ref
35-44y	28.0 (26.9 to 29.1)	0.87	-2.55 (-2.58 to -2.53)	0.10	164.8 (158.7 to 170.8)	0.16	87.5 (82.5 to 92.8)	0.14
45-54y	27.9 (27.0 to 28.8)	0.75	**-2.57 (-2.60 to -2.55)**	**0.013**	163.0 (158.2 to 167.7)	0.29	87.4 (83.0 to 92.0)	0.12
55-64y	**25.1 (24.2 to 26.0)**	**<0.001**	**-2.68 (-2.70 to -2.66)**	**<0.001**	161.7 (156.7 to 166.8)	0.51	**69.9 (65.2 to 75.0)**	**<0.001**
≥65y	**22.2 (20.8 to 23.5)**	**<0.001**	**-2.81 (-2.85 to -2.77)**	**<0.001**	162.3 (153.3 to 171.4)	0.56	**49.3 (44.1 to 55.1)**	**<0.001**
Education level								
Higher education	25.9 (25.3 to 26.6)	Ref	-2.60 (-2.62 to -2.58)	Ref	155.9 (152.0 to 159.8)	Ref	74.7 (71.4 to 78.1)	Ref
High school	**27.1 (26.4 to 27.8)**	**0.021**	-2.61 (-2.63 to -2.59)	0.49	**165.8 (161.7 to 169.8)**	**0.001**	**80.5 (77.0 to 84.2)**	**0.020**
No diploma	**27.7 (26.4 to 29.0)**	**0.020**	-2.63 (-2.66 to -2.60)	0.13	**174.1 (166.0 to 182.1)**	**<0.001**	**85.4 (77.7 to 93.9)**	**0.018**
Work type								
Sedentary	25.9 (25.2 to 26.6)	Ref	-2.61 (-2.63 to -2.59)	Ref	156.5 (152.5 to 160.6)	Ref	75.9 (72.6 to 79.3)	Ref
Standing	**27.9 (26.8 to 29.0)**	**0.002**	-2.63 (-2.66 to -2.60)	0.14	**180.5 (173.4 to 187.7)**	**<0.001**	**83.2 (77.7 to 89.1)**	**0.016**
Manual	**28.6 (26.6 to 30.7)**	**0.014**	**-2.67 (-2.70 to -2.63)**	**0.006**	**183.4 (173.7 to 193.1)**	**<0.001**	**86.0 (76.7 to 96.6)**	**0.043**
Not in paid employment	26.6 (25.5 to 27.7)	0.37	-2.59 (-2.62 to -2.57)	0.47	156.7 (150.7 to 162.6)	0.97	77.8 (72.8 to 83.2)	0.59
Mode of transport to work								
Motor vehicle	27.5 (26.9 to 28.1)	Ref	-2.57 (-2.58 to -2.55)	Ref	163.0 (159.5 to 166.6)	Ref	85.4 (82.3 to 88.6)	Ref
Public transport	27.9 (26.5 to 29.3)	0.57	-2.57 (-2.61 to -2.53)	0.74	166.1 (157.5 to 174.6)	0.51	91.1 (84.3 to 98.5)	0.14
Active travel	**30.0 (27.9 to 32.1)**	**0.025**	-2.54 (-2.59 to -2.49)	0.26	165.7 (155.0 to 176.3)	0.65	**102.4 (91.6 to 114.5)**	**0.002**
Smoking status								
Never	26.4 (25.9 to 27.0)	Ref	-2.62 (-2.63 to -2.60)	Ref	162.1 (158.8 to 165.4)	Ref	77.2 (74.3 to 80.1)	Ref
Former	26.9 (26.1 to 27.7)	0.33	**-2.58 (-2.60 to -2.56)**	**0.019**	161.4 (156.8 to 166.0)	0.80	81.2 (77.1 to 85.4)	0.12
Current	26.8 (25.5 to 28.2)	0.61	-2.64 (-2.67 to -2.61)	0.15	164.5 (156.6 to 172.3)	0.59	77.8 (70.9 to 85.4)	0.87
Self-rated health								
Very good	27.5 (26.7 to 28.3)	Ref	-2.57 (-2.59 to -2.55)	Ref	160.9 (156.8 to 164.9)	Ref	82.3 (78.6 to 86.2)	Ref
Good	**26.3 (25.7 to 26.9)**	**0.014**	**-2.63 (-2.64 to -2.61)**	**<0.001**	163.2 (159.7 to 166.6)	0.39	77.6 (74.6 to 80.8)	0.054
Fair	**25.3 (23.8 to 26.7)**	**0.008**	**-2.65 (-2.69 to -2.62)**	**<0.001**	162.3 (152.3 to 172.3)	0.79	**69.1 (62.1 to 76.9)**	**0.004**
Weight category								
Normal	27.5 (26.8 to 28.2)	Ref	-2.58 (-2.60 to -2.56)	Ref	165.0 (161.2 to 168.7)	Ref	82.0 (78.6 to 85.7)	Ref
Overweight	**26.1 (25.4 to 26.8)**	**0.005**	**-2.63 (-2.65 to -2.62)**	**<0.001**	162.6 (158.3 to 167.0)	0.42	77.1 (73.4 to 81.0)	0.073
Obese	**25.2 (24.2 to 26.3)**	**0.001**	**-2.65 (-2.68 to -2.63)**	**<0.001**	**153.5 (147.1 to 160.0)**	**0.004**	**70.7 (65.3 to 76.7)**	**0.002**

The data are weighted marginal means (95% confidence intervals). Bold font indicates statistically significant differences compared to the referent group (*p*<0.05)

*PA* Physical activity, *MVPA* Moderate-to-vigorous physical activity

The results of the logistic regression analysis showed that participants aged ≥65y were half as likely to report participating in sport or exercise relative to the youngest age group (OR (95% CI): 0.51 (0.28 to 0.95), *p*=0.032), as were manual compared to sedentary workers (0.50 (0.28 to 0.92), *p*=0.025). Poorer self-rated health (good: 0.43 (0.32 to 0.60), *p*<0.001; fair: 0.15 (0.09 to 0.26), *p*<0.001), overweight (0.61 (0.45 to 0.84), *p*=0.002) and obesity (0.47 (0.32 to 0.71), *p*<0.001) were all associated with lower odds of performing sport or exercise. Former smokers were more likely than never smokers to report participation in sports or exercise (1.66 (1.21 to 2.28), *p*=0.002). Full results of the logistic regression analysis are presented in Table S6. There were no differences in sport or exercise involvement by sex or education status. However, post-hoc Chi-square tests revealed that a higher proportion of women than men reported participating in water-based activities other than swimming, such as aqua-aerobics (9.1% versus 0.0%, *p*<0.001), and lower intensity activities such as yoga and Pilates (15.9% versus 3.0%, *p*<0.001). A higher proportion of men participated in racquet sports and ball sports (19.0% versus 5.7%, *p*<0.001). Running and hiking were more frequently reported by men of higher than lower educational status (no diploma: 11.5%; high school: 35.7%; higher education: 42.2%, *p*=0.013).

**Fig. 3:**
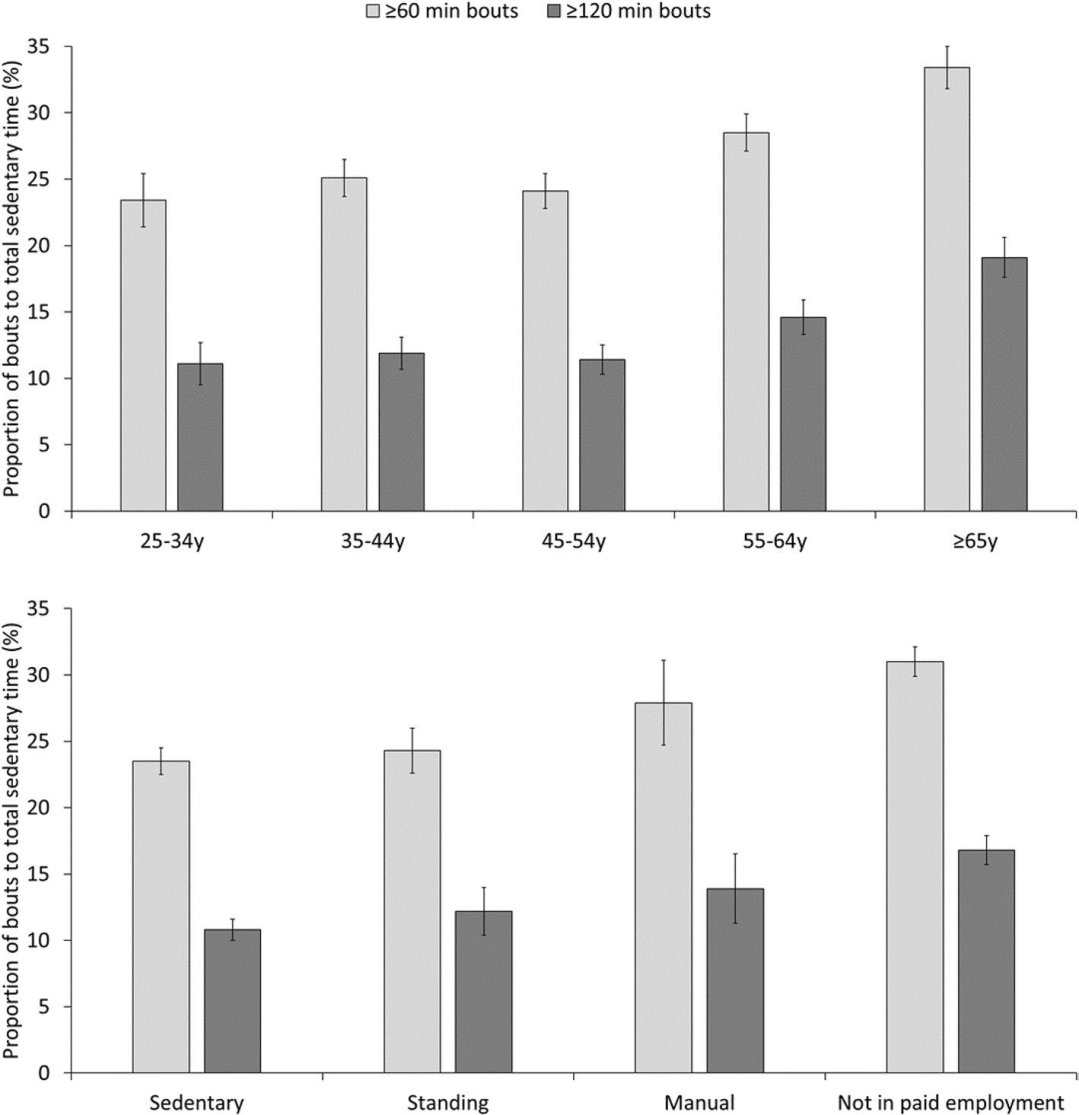
The proportion of total sedentary time accumulated in bouts of at least 60 and 120 min, stratified by age group (top) and work type (bottom). The data are weighted means and error bars represent 95% confidence intervals

Table 3 contains the marginal mean estimates of sedentary time and its accumulation pattern. Women performed less sedentary time, shorter bouts, and fewer bouts of prolonged sedentariness than men. Participants aged 55-64y and ≥65y performed more sedentary time and longer sedentary bouts compared to the youngest age group. Participants of lower educational attainment and normal weight status performed less sedentary time, shorter bouts, and fewer bouts of prolonged sedentariness. There was some indication that former smokers were less sedentary than never smokers, and that participants of fair self-rated health performed longer sedentary bouts than participants of very good health. Standing and manual workers accumulated less sedentary time and fewer bouts of prolonged sedentariness than sedentary workers, and standing workers performed shorter bout lengths. The results for power-law exponent alpha revealed that older age and not being in paid employment were both associated with a greater proportion of longer sedentary bouts. Accordingly, Fig. 3 illustrates that the proportion of total sedentary time accumulated in ≥60 and ≥120 min bouts was highest in the oldest age groups, and in participants who were not in paid employment. In the whole cohort, more than one-quarter (26.4%) of total sedentary time was accumulated in ≥60 min bouts, and 13.2% of time was accumulated in ≥120 min bouts. Tables S7-S8 provide the results stratified by men and women. There was substantial overlap in most confidence intervals. However, women aged 55-64y and ≥65y performed more prolonged sedentary bouts than the youngest women. Women who were not in paid employment were less sedentary and accumulated fewer prolonged sedentary bouts than women with a sedentary occupation. Traveling to work by public transport was associated with more sedentary time in men and with a greater proportion of longer sedentary bouts in women.

**Table 3:** Volume and pattern of total sedentary time, bout length, frequency and distribution

	Sedentary time (h/day)	***p***-value	Median bout length (min)	***p***-value	Number of prolonged bouts	***p***-value	PLE Alpha	***p***-value
Sex								
Men	12.5 (12.4 to 12.7)	Ref	32.4 (31.9 to 32.9)	Ref	5.9 (5.8 to 6.0)	Ref	2.45 (2.43 to 2.48)	Ref
Women	**11.7 (11.6 to 11.8)**	**<0.001**	**31.6 (31.2 to 32.1)**	**0.031**	**5.4 (5.2 to 5.5)**	**<0.001**	2.47 (2.45 to 2.49)	0.31
Age group								
25-34y	12.0 (11.8 to 12.2)	Ref	31.2 (30.3 to 32.0)	Ref	5.6 (5.4 to 5.8)	Ref	2.53 (2.49 to 2.57)	Ref
35-44y	11.9 (11.7 to 12.1)	0.64	31.2 (30.5 to 31.9)	0.93	5.5 (5.3 to 5.6)	0.31	2.49 (2.45 to 2.52)	0.085
45-54y	12.0 (11.8 to 12.2)	0.75	31.5 (30.9 to 32.1)	0.50	5.6 (5.5 to 5.8)	0.79	**2.47 (2.45 to 2.49)**	**0.031**
55-64y	**12.3 (12.1 to 12.5)**	**0.013**	**33.0 (32.3 to 33.7)**	**0.002**	5.8 (5.6 to 5.9)	0.25	**2.41 (2.38 to 2.44)**	**<0.001**
≥65y	**12.7 (12.4 to 13.0)**	**<0.001**	**34.2 (33.0 to 35.3)**	**<0.001**	5.8 (5.5 to 6.1)	0.29	**2.37 (2.33 to 2.41)**	**<0.001**
Education level								
Higher education	12.4 (12.2 to 12.5)	Ref	32.7 (32.2 to 33.2)	Ref	5.9 (5.8 to 6.0)	Ref	2.44 (2.42 to 2.47)	Ref
High school	**12.0 (11.9 to 12.1)**	**<0.001**	**31.5 (31.0 to 32.1)**	**0.003**	**5.5 (5.4 to 5.6)**	**<0.001**	2.48 (2.45 to 2.50)	0.051
No diploma	**11.7 (11.4 to 12.0)**	**<0.001**	**31.1 (30.1 to 32.0)**	**0.006**	**5.2 (4.9 to 5.4)**	**<0.001**	2.47 (2.43 to 2.51)	0.32
Work type								
Sedentary	12.3 (12.2 to 12.4)	Ref	32.0 (31.4 to 32.5)	Ref	5.9 (5.8 to 6.0)	Ref	2.48 (2.46 to 2.51)	Ref
Standing	**11.9 (11.7 to 12.1)**	**0.001**	**30.7 (29.9 to 31.4)**	**0.006**	**5.2 (4.9 to 5.4)**	**<0.001**	2.51 (2.47 to 2.55)	0.28
Manual	**11.6 (11.3 to 11.9)**	**<0.001**	32.2 (30.8 to 33.5)	0.80	**4.8 (4.6 to 5.1)**	**<0.001**	2.43 (2.36 to 2.50)	0.18
Not in paid employment	12.2 (12.0 to 12.4)	0.37	32.7 (32.0 to 33.4)	0.16	5.7 (5.5 to 5.9)	0.073	**2.42 (2.39 to 2.45)**	**0.003**
Mode of transport to work								
Motor vehicle	12.0 (11.9 to 12.1)	Ref	31.2 (30.7 to 31.7)	Ref	5.6 (5.5 to 5.7)	Ref	2.51 (2.49 to 2.53)	Ref
Public transport	12.3 (12.0 to 12.5)	0.090	31.5 (30.7 to 32.4)	0.51	5.8 (5.5 to 6.1)	0.24	2.48 (2.43 to 2.53)	0.28
Active travel	11.9 (11.5 to 12.2)	0.39	31.0 (29.7 to 32.4)	0.85	5.6 (5.2 to 6.0)	0.84	2.55 (2.48 to 2.61)	0.24
Smoking status								
Never	12.2 (12.1 to 12.3)	Ref	32.0 (31.6 to 32.5)	Ref	5.7 (5.6 to 5.8)	Ref	2.46 (2.44 to 2.48)	Ref
Former	12.0 (11.8 to 12.1)	0.068	31.9 (31.3 to 32.5)	0.69	5.6 (5.5 to 5.8)	0.83	2.46 (2.44 to 2.49)	0.92
Current	12.3 (12.1 to 12.6)	0.29	32.2 (31.2 to 33.1)	0.81	5.5 (5.3 to 5.8)	0.30	2.45 (2.40 to 2.49)	0.59
Self-rated health								
Very good	12.1 (12.0 to 12.3)	Ref	31.8 (31.3 to 32.3)	Ref	5.7 (5.6 to 5.8)	Ref	2.47 (2.45 to 2.50)	Ref
Good	12.1 (12.0 to 12.2)	0.72	32.0 (31.5 to 32.4)	0.59	5.6 (5.5 to 5.7)	0.27	2.46 (2.44 to 2.48)	0.36
Fair	12.3 (12.0 to 12.7)	0.33	33.0 (31.8 to 34.2)	0.089	5.7 (5.4 to 5.9)	0.85	2.44 (2.39 to 2.49)	0.29
Weight category								
Normal	12.0 (11.9 to 12.1)	Ref	31.6 (31.2 to 32.0)	Ref	5.5 (5.4 to 5.6)	Ref	2.47 (2.44 to 2.49)	Ref
Overweight	12.1 (12.0 to 12.3)	0.062	32.1 (31.5 to 32.6)	0.22	**5.7 (5.6 to 5.9)**	**0.034**	2.47 (2.44 to 2.49)	0.90
Obese	**12.6 (12.4 to 12.8)**	**<0.001**	**33.0 (32.0 to 34.0)**	**0.015**	**5.9 (5.7 to 6.1)**	**0.002**	2.44 (2.40 to 2.47)	0.19

The data are weighted marginal means (95% confidence intervals). Bold font indicates statistically significant differences compared to the referent group (*p*<0.05)

*PLE* Power law exponent

## Discussion

This is the first study to provide a comprehensive description of the levels of 24h acceleration, awake-time intensity-specific PA, and sedentary time in a population-based sample of adults living in Luxembourg. We highlight that, whilst adherence to recommended PA is high in Luxembourg, there is a considerable burden of sedentary time. In comparison to other studies, our estimates for the average 24h acceleration are lower than have been reported for middle-aged and older adults living in England (mean cohort age 50.7y: 31.4 m*g*) [22], and the wider UK (e.g. 31.2 m*g* in 45-54y olds) [23], but methodological inconsistencies prevent a direct (like-for-like) comparison of results. For instance, both comparator studies utilised the GENEActiv wrist-worn monitor, which registers ⁓10% higher magnitudes of acceleration relative to the Actigraph device [24]. The attachment site in the UK-wide study was also the dominant wrist, which records higher acceleration compared to devices worn on the non-dominant side [25]. Our estimate for the ≥65y age group closely aligns with values that are reported for older adults living in Spain (21.5 m*g*) [26] and England (⁓23.3 m*g*) [27]. However, the GENEActiv device was again used, which hinders comparability.

Multi-country studies that have implemented standardised questionnaires and methods across populations have consistently indicated that Luxembourg is one of the most active European nations [28–32]. Aligning with our estimate of 58% sports or exercise participation, a new report has shown that 63% of Luxembourgers perform weekly sport or exercise, a participation rate that is second only to Finland of 27 EU member states [33]. However, Luxembourg is simultaneously characterised by a relatively high prevalence of prolonged sitting [34, 35]. This is the first device-based study to show that the Luxembourg adult population spend on average more than 12 h/day sedentary, about 160 min/d in light PA, and 84 min/d in MVPA. Other device-based estimates captured across Europe typically show more daily time in light PA (ranging from 200 to 416 min/d), less sedentary time (7.3 to 10.2 h/day) and MVPA (26 to 69 min/d) [36–45]. Few investigations have reported equivalent or more daily MVPA [7, 46, 47]. It is important to note that sedentary time may have been underestimated in certain previous studies because accelerometers were often taken off in the evenings (in preparation for bed) and non-wear time was deleted. When likened to some investigations (conducted in older adults in Spain [26] and Switzerland [48], and similarly aged adults across Europe [49]) that included only 24h wear protocols, the daily time spent sedentary (11.4 to 12.7 h/day) is more closely comparable. Two studies of adults living in Sweden have similarly reported that a usual sedentary bout lasts on average for about 30 min [39, 40], but this is nearly twice as long compared to estimates for older adults living in England [50, 51]. In addition, we observed that more than one-quarter of total sedentary time was accumulated in ≥60 min bouts. The equivalent value has been estimated to be 6% in a multi-country sample of similarly aged Europeans [45] and about 10% in older adults living in Finland and England [38, 52]. The results highlight that long continuous sedentary bouts are prevalent in Luxembourg. Just over one-fifth of participants (21.9%) adhered to the former PA guideline for adults, which stipulated that ≥150 min MVPA/week must be accumulated in at least 10 min bouts. In stark contrast, the vast majority (98.1%) of Luxembourgers adhered to the new international PA guideline, which recommends the same activity volume but counts all incidental and short-length MVPA. High rates of adherence to the new PA guideline have also been reported in the UK (≥86%) [7, 46], Germany (100%) [47], and Finland (99.3%) [53].

In line with the majority of studies of European adults, we found that light PA was higher in women, and men accumulated more sedentary time and higher-intensity PA [7, 26, 37–48]. It is believed that men may participate in more leisure-time PA, including more sports, exercise and active recreational pursuits. Interestingly, we found no difference in overall participation, but there were sex differences in the types of leisure activities pursued. A higher proportion of men than women reported participating in racquet sports and ball sports. More women than men reported lower-intensity activities, such as yoga and Pilates, and water-based activities such as aqua-aerobics (swimming participation was equivalent: 17% involvement each). Accelerometers were asked to be removed whilst in the water, thus we may have underestimated higher intensity PA more so in women than men. Compared to the youngest age group, the average 24h acceleration and the time spent in MVPA were consistently lower and sedentary time profiles were least favourable in participants aged ≥55y. Several studies have reported marked adverse changes in movement behaviours beyond 50y [7, 23, 41–44]. Minimising unfavourable changes in movement behaviours in mid-life could have important implications for population health.

Investigations of European adults have shown that lower education is associated with less sedentary time and more time in light PA [26, 36, 43–47]. We found the same, and add to the literature showing that lower educational attainment is further associated with shorter sedentary bouts [26] and more time in MVPA [36, 44]. Some studies have reported that higher education is associated with more time in MVPA [39], particularly when it is expressed in bouts of at least 10 min [43, 46]. We found that the most educated men registered higher accelerations across the most active 15 and 30 min of the day. It is possible that people of higher educational status may have greater access to financial resources to fund leisure-time PA. They may also have more energy to be active outside of work due to less physically demanding jobs. Our self-reported data appear to support the latter, since men of higher educational status reported more running and hiking, neither of which requires prohibitively expensive equipment, gym or sports club membership.

Compared to a sedentary work type, standing and manual occupations were associated with a higher average 24h acceleration, and with more time in light PA and MVPA. Our estimates for manual work are likely underestimated, because accelerometers inadequately capture the increased energy costs of lifting, pushing, and carrying. A previous study advantageously combined chest acceleration with heart rate and found a much larger difference in MVPA between sedentary and manual workers in the UK [7]. It is important to note that there is uncertainty regarding the health benefits of occupational PA [54], and that we observed a steeper 24h intensity gradient (which corresponded to lower average accelerations across the most active 15 and 30 min of the day) in manual compared to sedentary workers. This is consistent with our observation that manual workers were 50% less likely to report sport or exercise participation compared to sedentary workers. As might be anticipated, having a sedentary occupation was associated with the least favourable sedentary profile, although not being in paid employment was associated with a greater proportion of longer sedentary bouts. There was some evidence in women, nonetheless, that not being in paid employment was also associated with less sedentary time, fewer bouts of prolonged sedentariness, and with a higher average 24h acceleration. This is likely due to sex differences in group membership. Most men who were not in paid employment were retired, whereas one-third of women were unpaid homemakers. Relative to commuting by motor vehicle there was some indication that traveling to work by public transport was associated with more sedentary time, a greater proportion of longer sedentary bouts, and with more time spent in MVPA in men. Studies have shown that public transport use is associated with more PA [55] but less so than active travel modes [56]. Likewise, we found more robust evidence that active travel was associated with a higher average 24h acceleration and with more time spent in MVPA. Our associations for commuting mode are similar in pattern and size to the results of a large scale analysis conducted using device-measured data in the UK Biobank study [57]. Free public transport was introduced across Luxembourg in March 2020. It will be essential to evaluate how this initiative has influenced commuting mode, national PA levels and sedentary patterns.

Smoking is inconsistently related to device-measured sedentary time and PA [7, 46–49]. We observed no associations between smoking status with the time spent in light PA or MVPA, but former smokers registered a shallower intensity gradient, higher average accelerations across the most active periods of the day, and a lower sedentary time than never smokers. A study of UK adults similarly reported that former smokers exhibited higher PA energy expenditure and more time in MVPA compared to never smokers [7]. This could be explained by the knowledge that smokers who are more physically active tend to have greater intention to quit [58]. Exercise is also promoted and advocated for smoking cessation, and since most smokers quit due to medical diagnoses or symptoms [59], they may be more likely to engage in PA as part of the clinical management of conditions. Post traumatic growth theory posits that trauma and adversity can lead to enduring positive psychological change, including re-evaluation and prioritisation of healthy lifestyle behaviours [60]. Consistent with this view, stopping smoking has been shown to predict more leisure-time PA over the long-term [61], and we found that former smokers were more likely to report sports or exercise participation than never smokers. Higher sedentary time and lower total and intensity-specific PA have consistently been reported as a function of higher weight status [7, 26, 42–48] and poorer health [36, 46]. In this study, higher weight status was one of the characteristics that was most consistently and least favourably related to outcomes. This may be due to reciprocal relationships between parameters (weight gain can promote physical inactivity, and vice versa) [62]. Poorer self-rated health was related to a lower average 24h acceleration and with less time in MVPA

### Implications

Luxembourg is a prominent financial centre and is the wealthiest country in the world per capita gross domestic product [10]. Its economy is largely based on international trade and banking, and more than two-thirds of working people in our sample were employed in sedentary occupations. These features may in part explain why more than half of every day appears to be spent sedentary in the Luxembourg adult population. Our finding that more than 98% of Luxembourg adults comfortably exceeded the new WHO PA guideline appears to be incompatible with the statistic that more than half of the population is overweight or obese [63]. This could be interpreted to mean that a higher weekly MVPA volume is warranted for population health, but most of our study sample (88.5%) exceeded even ≥300 MVPA min/week. The guideline volume of MVPA emerged mainly from evidence that was founded upon questionnaire-based studies about leisure-time PA [64], whereas removal of the 10 min bout requirement was based on evidence from device-measured data [65]. More accurate evidence from large device-based cohorts will be valuable in terms of refining the next iteration of guidelines. In view of the high adherence to currently recommended PA levels, and because long sedentary bouts that are harmful to health were highly prevalent, we suggest that breaking up continuous sedentary periods with active alternatives should be the focus of public health initiatives in Luxembourg. In particular, promoting light PA breaks could help to simultaneously reduce prolonged sedentariness and increase PA volume. Light PA is the largest contributor to PA energy expenditure [7], and replacing sedentary time with light PA has been shown to reduce cardiometabolic disease and mortality risk [8]. It is feasible to introduce light PA into daily routines and across domains, including the workplace without negatively affecting productivity [66]. It is also easier for many sections of the population to initiate light PA than more strenuous higher-intensity activity. We found that men aged ≥65y performed more light PA than the youngest men, and that time spent in light PA was unrelated to self-rated health status, which demonstrates that light PA is widely accessible. Encouraging participation in light PA can be used to help prepare individuals, who do not currently meet recommended MVPA levels*,* to participate in higher intensity PA. We highlight specific population strata that would benefit from replacing some sedentary time with PA.

### Strengths and limitations

This investigation benefitted from recent data collected in a large (relative to the total population size of Luxembourg) and well-characterised population-based sample of adults. This permitted a detailed description of movement behaviours across several factors, which we co-adjusted for in the analyses to identify independent associations with outcomes. We acknowledge that the ORISCAV-LUX 2 study sample is underrepresented with respect to younger and older ages and that participants were generally healthier than non-participants [12]. We used survey weights to generate nationally representative estimates, but this approach may not have alleviated the potential for selection biases. For instance, BMI was lower in our weighted sample compared to the whole ORISCAV-LUX 2 study population (26.3 kg/m^2^) and lower than estimates provided by the WHO (26.8 kg/m^2^) [67]. Reassuringly, adjusting our estimates to the average BMI of Luxembourgers based on WHO data reduced the time spent in MVPA by only 1.1 min/d. We advantageously utilised triaxial accelerometry and reproducible methods to provide an in-depth and comprehensive assessment of habitual movement behaviours, including investigation of raw accelerometer metrics, awake-time intensity-specific PA, sedentary time and its accumulation pattern. Participant compliance to the habitual activity assessment was excellent, and with the exception that monitors were asked to be removed whilst showering and swimming, the near-continuous (24h) wear protocol limited missing data and accompanying biases. These features enabled us to quantify adherence to the recommended weekly volume of PA rather than a supposed daily equivalent. It is a weakness, nonetheless, that wrist-worn accelerometers are prone to misclassifying standing behaviour as sedentary time, and we did not utilise a posture allocation algorithm to reduce misclassifications [68]. Additional studies should consider supplementing device-measured data with contextual information to explain movement patterns. This will be particularly valuable for discriminating between types of sedentary behaviour because certain modes may be advantageous for particular health outcomes. For instance, computer and internet use might benefit cognitive function in older adults [2].

## Conclusions

Adherence to the currently recommended weekly volume of MVPA is high in the Luxembourg adult population. However, half of all daily time is spent sedentary, and time in light PA is relatively low. Sedentary time and its accumulation pattern, and intensity-specific PA, vary across sociodemographic, employment, and health-related strata. We identify specific population subgroups that will benefit the most from targeted efforts to replace some sedentary time with PA.

## Supplementary Information

**Additional file 1: Table S1.** Patterns of whole day (24h) average acceleration and intensity distribution and awake-time intensity specific physical activity levels in men. **Table S2.** Patterns of whole day (24h) average acceleration and intensity distribution and awake-time intensity specific physical activity levels in women. **Table S3.** Patterns of Mx accelerations representing the acceleration above which the most active 480 min (one-third), 120 min, 60 min, 30 min, and 15 min of the day were accumulated. **Table S4.** Patterns of Mx accelerations representing the acceleration above which the most active 480 min (one-third), 120 min, 60 min, 30 min, and 15 min of the day were accumulated in men. **Table S5.** Patterns of Mx accelerations representing the acceleration above which the most active 480 min (one-third), 120 min, 60 min, 30 min, and 15 min of the day were accumulated in women. **Table S6.** Odds ratios for habitual performance of sport or exercise. **Table S7.** Volume and pattern of total sedentary time, bout length, frequency and distribution in men. **Table S8.** Volume and pattern of total sedentary time, bout length, frequency and distribution in women.

## Data Availability

De-identified data may be available upon reasonable request if consent is provided by all authors and the ORISCAV study group. Requests to access the data should be directed to LM.

## Abbreviations

BMI: Body mass index
CI: Confidence interval
MVPA: Moderate-to-vigorous physical activity
OR: Odds ratio
ORISCAV-LUX: Observation of cardiovascular risk factors in Luxembourg
PA: Physical activity
WHO: World Health Organization
